# Cognitive-behavioural therapy does not meaningfully reduce depression in most people with epilepsy: a systematic review of clinically reliable improvement

**DOI:** 10.1136/jnnp-2018-317997

**Published:** 2018-05-07

**Authors:** Adam J Noble, James Reilly, James Temple, Peter L Fisher

**Affiliations:** 1 Department of Psychological Sciences, University of Liverpool, Liverpool, UK; 2 Nidaros DPS, Division of Psychiatry, St. Olavs University Hospital, Trondheim, Norway

**Keywords:** epilepsy, depression, psychology, systematic reviews

## Abstract

Psychological treatment is recommended for depression and anxiety in those with epilepsy. This review used standardised criteria to evaluate, for the first time, the clinical relevance of any symptom change these treatments afford patients. Databases were searched until March 2017 for relevant trials in adults. Trial quality was assessed and trial authors asked for individual participants’ pre-treatment and post-treatment distress data. Jacobson’s methodology determined the proportion in the different trial arms demonstrating reliable symptom change on primary and secondary outcome measures and its direction. Search yielded 580 unique articles; only eight eligible trials were identified. Individual participant data for five trials—which included 398 (85%) of the 470 participants randomised by the trials—were received. The treatments evaluated lasted ~7 hours and all incorporated cognitive-behavioural therapy (CBT). Depression was the primary outcome in all; anxiety a secondary outcome in one. On average, post-treatment assessments occurred 12 weeks following randomisation; 2 weeks after treatment had finished. There were some limitations in how trials were conducted, but overall trial quality was ‘good’. Pooled risk difference indicated likelihood of reliable improvement in depression symptoms was significantly higher for those randomised to CBT. The extent of gain was though low—the depressive symptoms of most participants (66.9%) receiving CBT were ‘unchanged’ and 2.7% ‘reliably deteriorated’. Only 30.4% made a ‘reliable improvement. This compares with 10.2% of participants in the control arms who ‘reliably improved’ without intervention. The effect of the treatments on secondary outcome measures, including anxiety, was also low. Existing CBT treatments appear to have limited benefit for depression symptoms in epilepsy. Almost 70% of people with epilepsy do not reliably improve following CBT. Only a limited number of trials have though been conducted in this area and there remains a need for large, well-conducted trials.

## Introduction

Depression and anxiety are common comorbid conditions in people with epilepsy (PWE).^1^ Their presence is associated with an increase in suicide risk, healthcare costs, mortality and reduced quality of life.^2 3^ There are also knock-on effects for seizure control, with PWE who report symptoms of depression subsequently reporting poorer seizure control.^4^ Effective treatments are therefore required.

Psychotherapy, particularly cognitive-behavioural therapy (CBT), is recommended for the treatment of depression and anxiety in PWE.^3 5^ A Cochrane review^6^ and another meta-analysis^7^ concluded psychotherapy is efficacious for treatment of distress in PWE. However, the clinical relevance of the interventions is unclear as estimates of clinical significance were not included in the reviews.

There is increasing recognition that empirically supported interventions should at least partially be based on estimates of the clinical significance of the treatments.^8 9^ This would provide an index of absolute efficacy of psychological treatments. Furthermore, assessing the clinical significance of treatment effects would overcome the exclusive reliance on effect size (ES) estimates and allow multiple stakeholders to make more informed decisions. ESs may not reflect if the change was clinically meaningful while also obscuring within-group variability (including potential deterioration which has yet to be looked at). Funding bodies need to know about the clinical significance of current treatment effects to know whether investment is required to refine or develop new treatments. Policy and healthcare commissioners also need to know as it can help generate enthusiasm where necessary to alter practice and allow commissioners to better envisage the likely return from spend on one service compared with another.

The most established method for determining the clinical significance of a treatment is that of Jacobson and colleagues.^10^ It allows for the proportion of patients who deteriorate, remain unchanged and improve and recover having received a treatment to be determined. According to Jacobson’s criteria for recovery, an individual’s score on a trial’s primary outcome measure must meet two criteria: (i) the change in score from pre-treatment to post-treatment must be ‘statistically reliable’—that is, beyond that which can be accounted for by measurement error and (ii) the post-treatment outcome score must be in a range that renders them indistinguishable from a ‘well-functioning’ population. The approach can also be applied to the patients' scores on secondary outcome measures.

A notable feature of psychotherapy trials in epilepsy is the variability in the inclusion/exclusion criteria for the primary outcome variable.^7^ Some have not had explicit distress inclusion criteria; others have included PWE who did not demonstrate clinical distress (eg, see refs. 11 12). This may explain why the clinical significance of change in distress has been neglected. Because, as outlined above, to qualify as ‘recovered’, Jacobson’s criteria require the change to be sufficient to move a person from a ‘clinically unwell’ population to that of a ‘well’ population. Nevertheless, it is possible to assess if PWE satisfy Jacobson’s first criterion and achieve statistically reliable change (ie, ‘improvement’).

The present study therefore systematically identified trials evaluating psychotherapy for depression and/or anxiety in PWE and assessed their methodological quality. Individual patient data (IPD) were sought from authors for participants in their trials and the proportion of participants in each of the trials’ arms demonstrating ‘reliable improvement’, no reliable change and a ‘reliable deterioration’ on the trials' primary and secondary outcome measures for anxiety and depression was calculated. Risk differences (RDs) compared likelihood of participants making a ‘reliable improvement’ if they were in the trials’ psychotherapy arm rather than control arm.

## Methods

### Search strategy and selection criteria

The review was conducted according to Preferred Reporting Items for Systematic Reviews and Meta-Analyses guidelines. Medline, PsycINFO, PsycARTICLES, CINAHL plus, AHMED, clinicaltrials.gov, EThOS and SIGLE were searched from inception to March 2017 using Medical Subject Heading terms and keywords to identify suitable trials published in English (online supplementary table 1).

10.1136/jnnp-2018-317997.supp1 Supplementary file 1

To be eligible, trials had to meet the following criteria: (1) participants had to be PWE aged ≥18 years; (2) be randomly assigned to a psychological treatment or control condition and (3) a standardised measure of anxiety and/or depression had to be used as the primary outcome measure.

Titles and abstracts were reviewed by one author (JR). Excluded studies were independently reviewed by a second reviewer (PH-R; see Acknowledgements) against the inclusion criteria. There were no inconsistencies in judgement.

### Data extraction and study quality

The authors of eligible trials were contacted and raw data for participants on the trial’s outcome measures for psychological distress at pre-treatment and any follow-up points were requested, along with treatment allocations. The following information was extracted from the published articles using a standardised form: sample size and characteristics, eligibility criteria, treatment conditions, outcome measures, assessment points and attrition rates.

The methodological quality of trials providing IPD was assessed using the Physiotherapy Evidence Database (PEDro-P) Scale.^13^ Its first criterion addresses external validity, while criteria 2–11 address internal validity (online supplementary table 2). Each item is scored as yes (1) or no (0). Scores for items 2–11 are added to form an overall score: 9–10 indicates a methodologically ‘excellent’ trial, 6–8 ‘good’ quality, 4–5 ‘fair’ and ≤3 ‘poor’ quality.^14^

JR extracted the data and conducted the quality assessments. PH-R performed the same task on a random selection of 50%. There were no inconsistencies in judgement.

### Statistical analyses

Using the following formulae,^10^ applicable reliability statistics^15–20^ and the data summarised in table 1, a Jacobson Reliable Change Index (RCI) was calculated for each primary and secondary outcome measures of distress used by the trials providing IPD: $RCI=\frac{X_{2}-X_{1}}{S_{diff}}\;$ where $S_{diff}=\sqrt{2S_{E}^{2}}$ and $S_{E}=S_{1}\sqrt{\left(1-r_{xx}\right)}$

**Table 1:** Data used to determine the Reliable Change Index (RCI) for the primary and secondary outcome measures of distress in trials

Trial	Outcome measure/s		S_1_	r_xx_*	S_E_	S_diff_	RCI
Thompson *et al* (2010)^26^	Primary	mBDI^15^	12.51	0.88	4.33	6.13	12.00
	Secondary	PHQ-9^16^	7.06	0.89	2.34	3.31	6.49
Ciechanowski *et al* (2010)^27^	Primary	HSCL-20^17^	0.59	0.85	0.23	0.32	0.63
Schröder *et al* (2014)^12^†	Primary	BDI-I^18^	10.37	0.86	3.88	5.49	10.76
Gandy *et al* (2014)^11^†	Primary	NDDI-E^19^	3.59	0.85	1.39	1.96	3.85
		HADS-D^29^	4.00	0.82	1.70	2.40	4.70
	Secondary	HADS-A^29^	3.76	0.83	1.55	2.19	4.29
Thompson *et al* (2015)^22^	Primary	mBDI^15^	9.42	0.88	3.26	4.62	9.05
	Secondary	PHQ-9^16^	3.63	0.89	1.20	1.70	3.34

The designation of outcome measures as being the primary or secondary outcome measure was taken directly from trial reports, except for Gandy *et al* (2014)^11^ (see Outcome assessment section).

*Internal consistency for: mBDI,^15^ PHQ-9,^16^ HSCL-20,^17^ BDI-I,^18^ NDDI-E,^19^ HADS-D and HADS-A.^20^

†The extent of reliable change in and across the trials was also calculated when participants from the trials who did not have a score indicative of clinically significant depression at baseline were excluded. There were such participants in only the Schröder *et al* 12 (2014) (n=3 intervention and n=5 control participants) and Gandy *et al* 11 (2014) (n=9 intervention and n=9 control participants) trials. The exclusion of these participants meant the individual RCIs for these two trials needed to be recalculated as the SD of the trial samples on the primary outcome measure of interest for the trial changed. The recalculaed RCI was 9.23 for Schröder *et al* 12 (2014) and 2.41 for Gandy *et al* 11 (2014).

BDI, Beck Depression Inventory; HADS-A, Hospital Anxiety and Depression Scale–Anxiety subscale; HADS-D, Hospital Anxiety and Depression Scale–Depression subscale; HSCL-20, Hopkins Symptom Checklist-20; mBDI, modified Beck Depression Inventory; NDDI-E, Neurological Disorders Depression Inventory for Epilepsy; PHQ-9, Patient Health Questionnaire-9; r_xx_, reliability of the scale; S_1_, SD at pre-treatment; S_diff_, SE of difference; S_E_, SE of measurement. Individual patient data for the secondary outcome measure NDDI-E within Thompson *et al* 22 (2015) were not made available for analysis.

X_1_ is the pre-treatment score of an individual and X_2_ is the post-treatment score of an individual.

The RCI identifies the threshold beyond which symptoms must change on an outcome measure for it to be considered reliable. An RCI greater than ±1.96 was required for the change to qualify as statistically reliable at P<0.050.^10^

The earliest assessment following completion of the treatment within each trial was considered the post-treatment outcome assessment. On average, these took place <2 weeks after treatment had finished.

The proportions of participants in each trial arm demonstrating ‘reliable improvement’, no reliable change and a ‘reliable deterioration’ at the post-treatment assessment on each of the outcome measure were calculated along with 95% confidence intervals (95% CIs). All analyses were completed on an intention-to-treat basis.

For each trial, a RD (and 95% CI) was calculated by subtracting the incidence of ‘reliable improvement’ in the trial’s control group from that seen in the treatment group on its outcome measures. The RDs for the separate trials on their primary depression outcome measure were then pooled using the Mantel-Haenzel random effects model. RDs for the secondary outcome measures were pooled in a similar way.

To explore possible treatment moderators, we calculated the pooled RD for reliable improvement on the primary outcome measures for those trials evaluating individual face-to-face CBT and compared this with the pooled RD for trials evaluating other forms of CBT. We also calculated the pooled RDs for these two sets of treatments when participants from the trials who did not have depression at baseline were excluded. The latter required recalculation of the RCI for some trials (see table 1 footnote).

Not all eligible trials provided IPD. To permit a comparison of the effects of the psychological treatments in trials that did and did not provide IPD, standardised mean difference (SMD) ESs (Hedges' g) and 95% CIs were calculated for the primary outcome measures for all eligible trials. SMDs for trials providing IPD were pooled using an inverse variance random effects model and statistically compared with the pooled SMD for trials not providing IPD. The following guidelines can be used for interpreting SMDs: 0.2=small, 0.5=medium and 0.8=large.^21^

All analyses were conducted using CMA 3.3 (Comprehensive Meta-Analysis, Biostat, Englewood, New Jersey, USA).

## Results

### Study selection

#### Search results and IPD received

**Figure 1:**
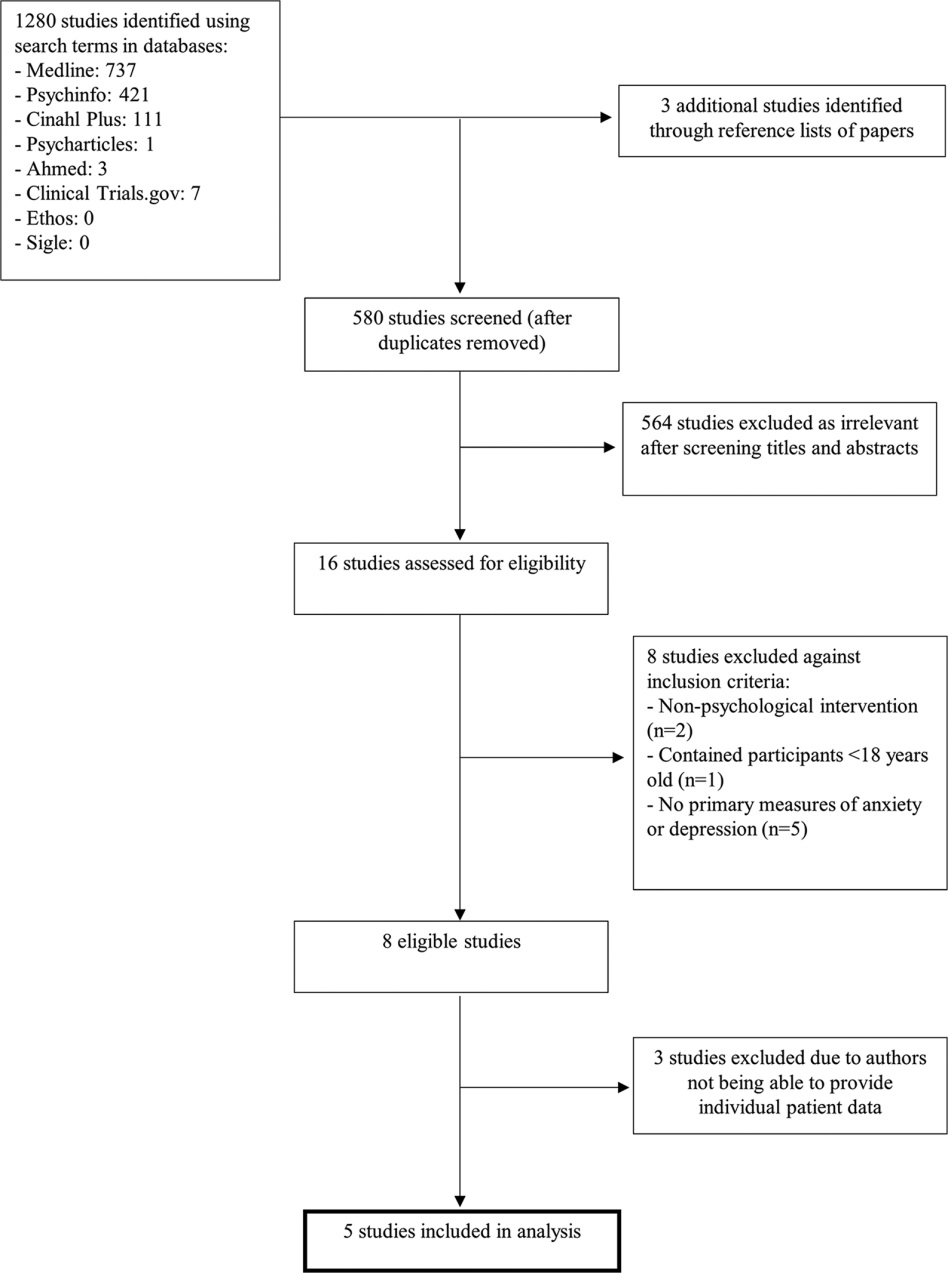
Preferred Reporting Items for Systematic Reviews and Meta-Analyses diagram summarising the screening process for the included studies.

The search yielded 1283 articles (figure 1); 580 remained after duplicates were removed. After a title and abstract review, 16 full-text articles were retrieved and assessed for eligibility. Eight trials were eligible,^12 22–27^ with the results of one trial being reported across two articles.^27 28^Online supplementary table 3 describes their characteristics.

IPD were made available for five of the eligible trials—namely, Thompson *et al,* 26 Ciechanowski *et al,* 27 28 Schröder *et al,* 12 Gandy *et al* 11 and Thompson *et al,* 22 The features of these trials are reported below.

The three trials not able to provide IPD (Davis *et al,* 23 Tan and Bruni^24^ and McLaughlin and McFarland^25^) tended to be older (mean year of publication 1993 vs 2013) and have smaller starting samples than trials providing IPD (mean 24 vs 80).

#### Design of trials

Of the trials providing IPD, three employed parallel-group designs,^11 12 27^while Thompson *et al*’s two trials^22 26^ employed cross-over designs. The ‘interim’ assessments in the latter constituted the post-treatment outcome assessments.

#### Treatment conditions

All five trials evaluated a psychological therapy which involved at least an element of CBT. Three used individual treatment (treatment was face to face in two trials and online in one) and two group treatment (delivered by either phone or internet in the two trials). The exact components of the interventions are detailed in online supplementary table 3.

On average, the intended duration of the treatments was 7.33 hours (SD=0.94, range 6–8; excluding homework and subsequent monitoring) and involved a mean of 8.8 sessions (SD=1.79, range 8–12). The extent to which participants in the trials actually attended planned sessions was reported by four trials and largely high. None of the trials, however, reported information on treatment fidelity.

#### Outcome assessment

On average, participants’ post-treatment outcome assessments occurred 12 weeks following randomisation. This was, on average, 1.7 weeks (SD=2.1, range 0–5) after treatment had finished.

The primary outcome measures used by all the trials were self-report depression questionnaires. The specific instruments used are reported in online supplementary table 3. It should be noted that Gandy *et al* 11 used both the Neurological Disorders Depression Inventory for Epilepsy (NDDI-E)^19^ *and* the depression subscale of the Hospital Anxiety and Depression Scale,^29^ but did not specify which was primary. We considered the NDDI-E to be the primary measure.

Three trials^11 22 26^ included secondary outcome measures of depression and one^11^ a measure of anxiety. All used standardised instruments.^16 29^ We also calculated the direction and reliability of change participants demonstrated on these secondary measures.

#### Participants

Across the five trials, 398 PWE were randomised. The weighted mean age of participants was 39.9 years and most (66.6%) were women (range within trials 52.5–81.1%). Due to loss to follow-up, pre-treatment and post-treatment IPD were secured for 315 (79.1%) of these participants. The proportion of participants providing post-treatment outcome data within the trials ranged from 73.1% to 84.4%.

Information provided by trials on the epilepsy characteristics of their samples varied and was not reported in a standardised way. For example, in terms of the frequency and recency of seizures, Thompson *et al* 26 reported 76% of their participants had a seizure (undefined) in the prior 4 weeks and Ciechanowski *et al* 27 said 74% of their participants had a seizure (any type permitted) in the prior 6 months, while Gandy *et al* 11 reported 48% of their participants had neurologist judged ‘refractory’ epilepsy, but provided no criteria for this.

Most participants in the trials demonstrated clinically relevant depression at their pre-treatment assessment (mean=89.2%; range 60–100%). There was though variability in the initial inclusion criteria adopted by the trials for distress: two^22 26^ used the Center for Epidemiological Study of Depression measure^30^ to identify depression, but used different cut-offs; one^12^ required participants to informally report having experienced depressive symptoms (but specified no time period); another^27^ included those who at interview demonstrated dysthymia, as well as those with a score on the Patient Health Questionnaire-9^16^ indicative of depression. The final trial^11^ did not require participants to have any depressive symptoms to be eligible.

In all five trials, treatment and control group participants were reported to be comparable in pre-treatment distress.

#### Trial quality

Trial quality is reported in online supplementary table 2. As all trials specified eligibility criteria, they each scored one for external validity. The average internal validity score was 6.2/10 (range 5–7). All studies used random allocation methods and concealed treatment allocation, but only two used blinded outcome assessors^12 27^ and all had an attrition rate >15% at their primary outcome assessment point.

### Effects of interventions

#### Reliability and direction of change on primary outcome measures

Following psychological treatment, the extent of depressive symptoms experienced by most (66.9%) participants did not reliably change (trial range 46.9–75.0%); 30.4% of participants made a ‘reliable improvement’ (range 21.2–50.0%) and 2.7% made a ‘reliable deterioration’ (range 0–5.3%) (table 2). This compared with 83.2%, 10.2% and 6.6%, respectively, for the controls.

**Table 2:** Classification of change in participants’ psychological distress in individual trials between pre-treatment and post-treatment assessment according to Jacobson’s Reliable Change Index

Trial	Reliable change category (%)			
	n	Deteriorated	Unchanged	Improved
*Thompson et al* (2010)^26^				
*Primary*: mBDI				
CBT+M	19	5.3	63.2	31.6
WLC	21	4.8	71.4	23.8
		Difference in % improved relative to control=7.8		
*Secondary:* PHQ-9				
CBT+M	19	10.5	57.9	31.6
WLC	21	9.5	71.4	19.0
		Difference in % improved relative to control=12.6		
*Ciechanowski et al* (2010)^27^				
*Primary:* HSCL-20				
CBT	32	3.1	46.9	50.0
TAU	33	12.1	63.6	24.2
		Difference in % improved relative to control=25.8		
*Schröder et al* (2014)^12^				
*Primary:* BDI-I				
CBT+M + ACT	25	0	72.0	28.0
WLC	32	0	96.9	3.1
		Difference in % improved relative to control=24.9		
*Gandy et al* (2014)^11^				
*Primary:* NDDI-E*				
CBT	20	0.0	75.0	25.0
WLC	25	12.0	80.0	8.0
		Difference in % improved relative to control=17.0		
*Secondary:* HADS-D				
CBT	20	5.0	85.0	10.0
WLC	25	4.0	92.0	4.0
		Difference in % improved relative to control=6.0		
*Secondary:* HADS-A				
CBT	20	5.0	80.0	15.0
WLC	25	8.0	80.0	12.0
		Difference in % improved relative to control=3.0		
*Thompson et al* (2015)^22^				
*Primary:* mBDI				
CBT+M	52	3.8	75.0	21.2
WLC	56	5.4	92.9	1.8
		Difference in % improved relative to control=19.4		
*Secondary:* PHQ-9				
CBT+M	52	17.3	69.2	13.5
WLC	56	19.6	75.0	5.4
		Difference in % improved relative to control=8.1		

Duration of intervention and timing of post-treatment assessment were: Thompson *et al* (2010)^22^ intervention duration= 8 weeks, timing of post-treatment assessment= ~8 weeks; Ciechanowski *et al* 27 (2010) intervention duration= 19 weeks, timing of post-treatment assessment=24 weeks; Schröder *et al* 12(2014) intervention duration= 9 weeks, timing of post-treatment assessment= 9 weeks; Gandy *et al* (2014)^11^ intervention duration= 8 weeks, timing of post-treatment assessment= 9 weeks and Thompson *et al* (2015)^22^ intervention duration= 8 weeks, timing of post-treatment assessment= 10.5 weeks. Individual patient data for the secondary outcome measure NDDI-E within Thompson *et al* 22 (2015) were not made available for analysis.

*The designation of outcome measures as being the primary or secondary outcome measure was taken directly from trial reports, except for Gandy *et al* (2014)^11^ (see Outcome assessment section).

ACT, Acceptance and Commitment Therapy; BDI, Beck Depression Inventory; CBT, cognitive-behavioural therapy; HADS-A, Hospital Anxiety and Depression Scale–Anxiety subscale; HADS-D, Hospital Anxiety and Depression Scale–Depression subscale; HSCL-20, Hopkins Symptom Checklist-20; M, mindfulness; mBDI, modified Beck Depression Inventory; NDDI-E, Neurological Disorders Depression Inventory for Epilepsy; PHQ-9, Patient Health Questionnaire-9; TAU, treatment as usual; WLC, waitlist control.

**Figure 2:**
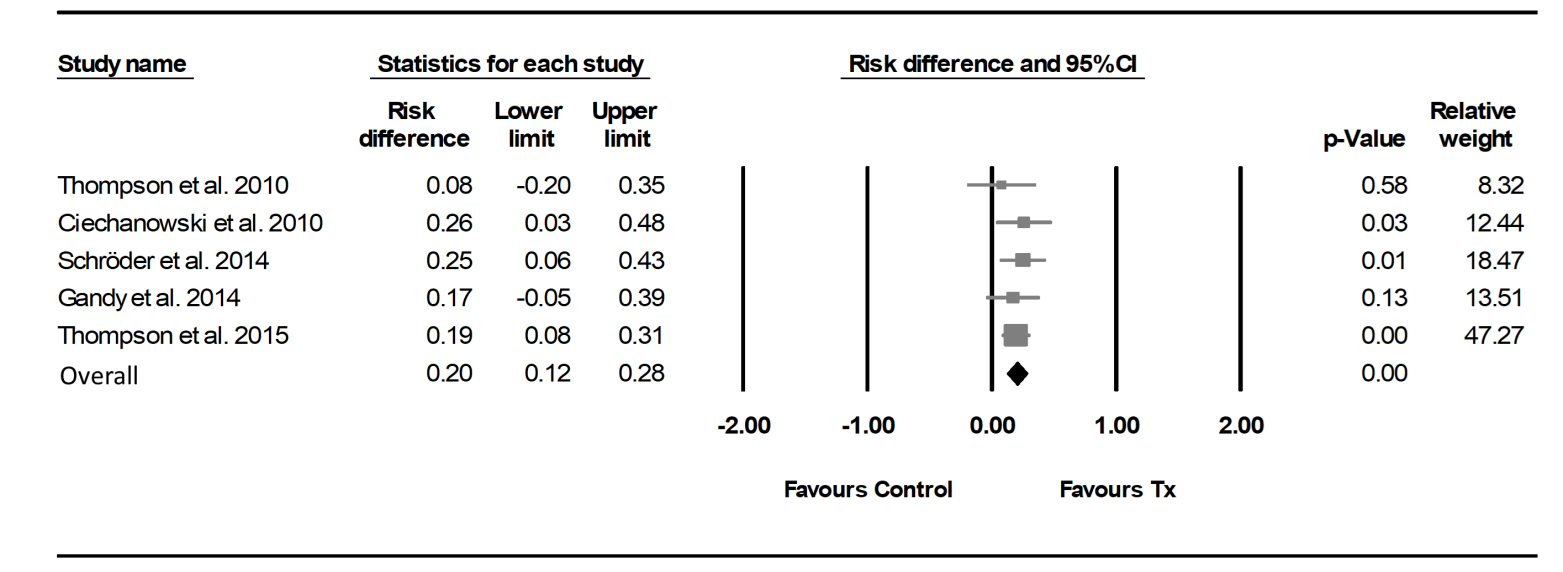
Difference in proportion of participants showing ‘reliable improvement’ within eligible trials on primary outcomes of depression post-treatment. Duration of intervention, timing of post-treatment assessment and primary outcome measure used were: Thompson *et al* (2010)^22^ intervention duration=8 weeks, timing of post-treatment assessment=~8 weeks, measure=modified Beck Depression Inventory (mBDI-I); Ciechanowski *et al* (2010)^27^ intervention duration=19 weeks, timing of post-treatment assessment=24 weeks, measure=Hopkins Symptom Checklist-20 (HSCL-20); Schröder *et al* 12 (2014) intervention duration=9 weeks, timing of post-treatment assessment=9 weeks, measure=Beck Depression Inventory (BDI-I); Gandy *et al* 11 (2014) intervention duration=8 weeks, timing of post-treatment assessment=9 weeks, measure=Neurological Disorders Depression Inventory for Epilepsy (NDDI-E) and Thompson *et al* (2015)^22^ intervention duration=8 weeks, timing of post-treatment assessment=10.5 weeks, measure=mBDI. Please note that the designation of outcome measures as being the primary or secondary outcome measure was taken directly from trial reports, except for Gandy *et al* (2014)^11^ (see Outcome assessment section).

For three trials,^12 22 27^ the RD indicated the likelihood of making a ‘reliable improvement’ was statistically higher in the psychological treatment arms (figure 2). The pooled RD across the five trials was 0.20 and statistically significant (95% CI 0.12 to 0.28; Q=1.34, df 4, P=0.85). Thus, on average, 20% *more* participants in the intervention groups showed a ‘reliable improvement’ in their experience of depressive symptoms compared with the control conditions.

The trial in which intervention participants were most likely to demonstrate ‘reliable improvement’ was Ciechanowski *et al*,^27^ with 25.8% more of their intervention participants showing a ‘reliable improvement’ compared with the control condition (RD=0.26; 95% CI 0.03 to 0.48).

The pooled RD for reliable improvement was only slightly higher for trials evaluating individual face-to-face CBT (pooled RD=0.21, 95% CI 0.06 to 0.37) compared with trials evaluating other CBT forms (pooled RD=0.19, 95% CI 0.10 to 0.29). Following individual face-to-face CBT treatment, 40.4% of participants made a ‘reliable improvement’ compared with 17.2% of controls. Following other forms of CBT, 25.0% of participants improved compared with 6.4% in the controls (online supplementary figure 1).

When those without clinically significant depression at baseline were excluded from analyses, the pooled RD for the five trials remained at 0.20 (95% CI 0.11 to 0.29). The pooled RD for the trials evaluating individual face-to-face CBT did though increase to 0.29 (95% CI 0.10 to 0.48). Its CI, however, overlapped with the pooled RD for the other forms of CBT (pooled RD=0.18, 95% CI 0.08 to 0.27) (online supplementary figure 2). Individual face-to-face CBT treatment here led to 51.2% of participants making a ‘reliable improvement’ compared with 22.5% of controls, while other forms of CBT were associated with 25.8% of participants improving compared with 9.6% of the controls.

#### Reliability and direction of change on secondary outcome measures

When the analysis was restricted to scores on the secondary outcome measures of depression used by Gandy *et al*,^11^ Thompson *et al* 26 and Thompson *et al*,^22^ only 8% more participants in the psychological treatment groups made a ‘reliable improvement’ relative to the trials’ control conditions (pooled RD=0.08, 95% CI −0.01 to 0.16) (table 2). This is lower than indicated by the primary outcome measures used by these three trials (pooled RD=0.18, 95% CI 0.08 to 0.27).

Gandy *et al* 11 included the only measure of anxiety. Being in the psychological treatment arm in that trial was *not* associated with a significantly greater likelihood of ‘reliable improvement’ (RD=0.03, 95% CI −0.17 to 0.23); 15% of the treatment group made a ‘reliable improvement’ in anxiety symptoms compared with 12% of controls.

#### Effect sizes

**Figure 3:**
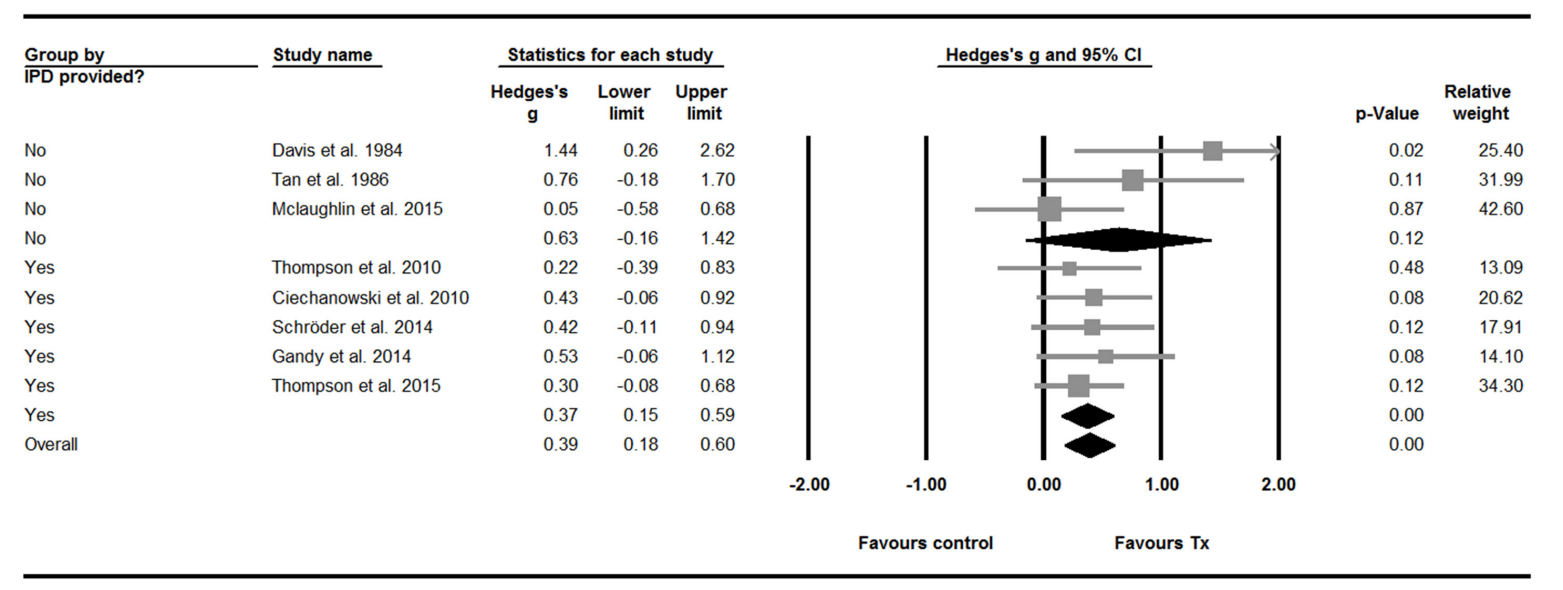
Effect sizes (ESs) in individual trials on primary outcome measures of depression—pre–post-treatment change. For trials providing individual patient data (IPD), ESs were calculated using IPD pre–post mean change and pre–post SD difference. For the trials that did not, aggregate statistics available within the published articles were used. For McLaughlin and McFarland (2015), the ES was calculated based on aggregate data pre–post mean change and pre–post SD differences. For Davis^23^ *et al* (1984) and Tan and Bruni^24^ (1986), ESs were calculated on the basis of independent groups t-test estimated P values and post-test means. Davis^23^ *et al* (1984) reported that P<0.1, so 0.1 was used as a conservative estimate. Tan and Bruni^24^ (1986) reported that P<0.05, so 0.6 was be used as a conservative estimate.

The ESs for the five trials providing IPD show that each of the psychological treatment they evaluated was associated with a greater reduction on the primary outcome measure for depression. None, however, reached statistical significance (figure 3). The pooled SMD for these trials did reach statistical significance and indicated a small effect favouring the intervention: 0.37, 95% CI 0.15 to 0.59. A subgroup comparison did not find this pooled SMD to be significantly different from the pooled SMD for the three trials^23–25^ not providing IPD (Q=0.39, df 1, P>0.05). The pooled SMD for the latter was larger, but had a wide CI and did not indicate a statistically significant effect: 0.63, 95% CI −0.16 to 1.42.

## Discussion

### Summary of results

Psychological therapies, including CBT, have been recommended for the treatment of depression and anxiety in PWE.^3 5^ Such recommendations were arguably premature as the extent to which they afford patients clinically relevant reductions in symptomatology was unknown. We systematically identified relevant trials and determined the proportion of PWE demonstrating ‘reliable improvement’ in distress levels following treatment and compared it with that seen in control conditions.

Our findings are revealing. All trials evaluated treatments that focused on or incorporated elements of CBT. Overall, these CBT treatments did lead to PWE being more likely to show improvement. However, the proportion of patients making an improvement was low. Across the trials, only ~30% of PWE who received the treatments showed a reliable improvement in their depressive symptoms. Despite receiving ~7 hours of treatment, depressive symptoms for the remaining 70% of participants remained largely unchanged. On average, 20% *more* participants in the intervention groups made a reliable improvement compared with control conditions. This increase in the proportion of patients making an improvement is important. However, as we detail below, we contend higher rates of improvement are needed.

The clinical effectiveness of CBT treatment for anxiety (which in PWE often revolves around seizure worry, medication side effects and stigma) remains unknown. Only Gandy *et al* 11 evaluated the effect of such treatments on anxiety and there it was a *secondary* outcome measure. They found 15% of participants receiving treatment made a reliable improvement—only 3% more than when no treatment was given.

### The clinical utility of available psychological treatments

What proportion of PWE experiencing depression/anxiety would need to demonstrate a reliable improvement for an intervention to be classed as effective? This is a value judgement, but it seems reasonable to conclude that when only 30% of treated patients make an improvement that highly effective CBT treatments for distress in epilepsy do not exist. When the same criterion has been used to evaluate psychological treatments for major depression, obsessive compulsive disorder and generalised anxiety disorder outside of epilepsy, treatments have there been able to demonstrate an ability to elicit much higher rates of reliable improvement (ie, ~50–80%).^31–33^

Indeed, our study may provide an inflated estimate of CBT’s benefit. We could only determine the proportion of participants demonstrating a change score that was unlikely to be attributable to measurement error. It was not possible to apply the second and more stringent of Jacobson *et al*’s^10^ criteria and determine whether the change meant the person also returned to normal functioning. It is possible some individuals classified as having made a reliable improvement, especially those with severe pre-treatment distress, may have remained symptomatic. Loerinc *et al* 34 examined response to CBT for anxiety in the general population. The proportion classed as having responded was 28% lower when ‘response’ was defined as needing to satisfy *both* of Jacobson *et al* 10 criteria, rather than just one.

Our study may also overestimate the benefits afforded to PWE from CBT when used in naturalistic settings. Trials typically use intensively trained and supervised therapists. They also have strict participant inclusion/exclusion criteria (eg, no suicidal ideation and no cognitive impairment). A sizeable proportion of participants in the trials also did not experience frequent seizures. More frequent and/or severe seizures would likely reduce treatment response by disrupting an individual’s ability to attend and engage in therapy sessions, homework and take on information. Another factor potentially influencing our results is publication bias.

Our conclusion regarding the utility of available CBT treatments is somewhat at odds with that of the latest Cochrane review.^6^ That review’s primary focus was on the effect of psychological treatments on quality of life in epilepsy. As a secondary objective, it did though consider the effect of the interventions on depression and anxiety. It concluded beneficial psychological treatments *do* exist and did not recommend the need for more effective treatments. Our reviews used different methods. However, might it also be that too low a threshold has previously been set before it is concluded effective treatments exist?

The evidence that led to Michaelis *et al*’s^6^ conclusion was psychotherapy demonstrated relative efficacy in reducing depression symptoms in 7/11 studies, and receipt of psychotherapy and other psychological interventions was associated with an improvement in quality of life in 3/11 studies. The improvement was though marginal. When measured using the Quality of Life in Epilepsy Inventory-31,^35^ which has a score range of 0–100, the effect equated to just a 5-point increase. We contend such findings are not sufficient to conclude *effective* treatments exist, rather that there is substantial room for improvement. To conclude otherwise risks us failing to stimulate efforts to develop more effective treatments.

### Why were the treatments largely ineffective?

Although questions about its utility remain, CBT is the recommended treatment for depression in the general population.^36^ So why did it not benefit most PWE?

Possible explanations are the treatments within the trials were not delivered well and participants did not receive the full treatment ‘dose’. We cannot rule this out. Fidelity was not reported and we were only able to complete analyses on an intention-to-treat basis. The efficacy of the treatments for people who completed treatment might therefore be underestimated. Most trials did though say treatment delivery was closely supervised and most participants attended most treatment sessions.

Might it also be that PWE with so-called ‘treatment-resistant depression’ (TRD) were over-represented in the trials and this attenuated the effect of psychotherapy? This appears unlikely; none of the trials reported the presence of TRD in their samples and only ~30% of participants across the trials were noted to be on or previously been on an antidepressant.

A more radical explanation is the CBT treatment approach is not suitable for PWE. It has been taken as a given that the assumptions about distress underlying this treatment apply to PWE. This may not be the case. The cognitive model,^37^ which informs CBT, proposes people vulnerable to depression have depressogenic schemas about themselves, the world and future. Activated by stressful events, these give rise to biased thinking and unrealistic cognitive appraisals of events which in turn negatively affect feelings and behaviour. One way CBT seeks to reduce depression is by helping people identify and challenge unhelpful cognitions, so more realistic and balanced appraisals can be elicited. How though would this work for PWE? The thoughts and concerns PWE say they are troubled by can be realistic (eg, ‘my seizures can happen at any time’, ‘my medication has side effects’, ‘society treats me differently’ and ‘epilepsy puts a strain on my family’). Identifying and trying to modify these thoughts may therefore offer PWE little relief.

Evaluating alternative psychotherapeutic approaches, particularly those that do not view dysfunctional attitudes as being central to the development or maintenance of distress, might thus be warranted. One candidate is metacognitive therapy (MCT).^38^ The central tenets and predictions of the model underlying it have been found to hold in epilepsy^39 40^ and, importantly, increasing evidence within the mental health literature shows MCT is effective in treating anxiety and depression, superior to waitlist controls, and possibly CBT.^41^

### Implications

Our findings indicate that while currently available CBT treatments do not harm patients (at least not according to measures considered and the cut-off we applied), they are seemingly not as effective as might have been thought. What should therapists therefore tell PWE who, in the short term, will continue to be offered such treatments?

Within most healthcare settings, professional and ethical standards mean clinicians are obliged to disclose the risks and benefits of treatments offered. Views among psychotherapists about the importance, and feasibility, of informed consent do though vary.^42^ Some might have concerns that sharing the findings from our study at the outset may diminish treatment effects by reducing patients’ expectations. Whether this would happen remains to be determined. Moreover, it is worth considering the consequences of not providing accurate information about likely treatment response.

Psychotherapy requires substantial investments of effort and time on the patient’s part, often in the context of competing demands. During therapy orientation, they will be instructed as to what is expected of them, including homework. Given such a preface, some patients might attribute lack of treatment response to a failure on their behalf, compounding feelings of helplessness and low self-esteem. Alternatively, patients might, having invested their time with little return, feel misled, see the experience as negative and be less inclined to accept subsequent treatments. Crawford *et al* 43 surveyed >14 000 UK adults who had psychological therapy (typically CBT). Those who said they had been given sufficient information about their treatment were less likely to report negative effects.

We would acknowledge at this point though that the generalisability of our results to clinical practice is unclear. Most interventions evaluated by trials in our review resemble ‘brief’, rather than ‘standard CBT’—that is, ~8 sessions in length, rather than 10–20.^44 45^ It is unclear how many sessions clinicians are offering PWE in practice. It is the case though that epilepsy guidelines recommending CBT do not specify session length and the trials which they contend support CBT’s use are mostly the same ones that are in our review. This may mean clinicians are offering brief CBT. Further raising this possibility are bodies such as the UK’s National Institute for Health and Care Excellence^46^ support the use of brief CBT for depression in those with chronic physical health conditions (at least as first-line treatment for mild-moderate depression—which was the typical level seen in the PWE who participated in the trials in our review).

Our findings also have implications for future research. While the methodological quality of the trials was judged to be ‘good’, areas for improvement existed. Not all trials used treatment-blind outcome assessors and loss to follow-up was high. Since methodologically weak trials overestimate treatment effects, future trials should seek to address these limitations. It is important to highlight though that while used in a previous review in the area,^7^ the generic quality assessment tool we used is unlikely to give a full account of the trials’ methodological rigour. It, for example, penalised the trials for not blinding participants and therapists, despite this being almost impossible to achieve in a psychotherapy trial. At the same time, however, the scale did not evaluate the trials against criteria uniquely relevant to psychotherapy trials—such as whether the intensity of the control and treatment condition matched, whether the treatment was sufficiently described and replicable, whether the therapist was sufficiently trained and whether treatment delivery adhered to the manual and was competent.

What was also apparent was the extent of participant description was poor and non-standardised—not only does this limit evaluations as to how representative samples are, but it means it is difficult to begin to identify possible treatment moderators. Future trials should follow available reporting guidelines.^47^ It would also be helpful if future trials incorporate health economic evaluations, so the cost-effectiveness of treatments can be determined.

### Strengths and limitations

This study is the first analysis of its kind in the field and has several strengths. First, the criteria used to see how meaningful the change was were standardised and empirically based. Minimal attention has previously been given to the issue and idiosyncratic and arbitrary criteria had been used. Second, as well as considering change on the trials’ primary measures, we also calculated and considered change on secondary outcome measures. This confirmed that improvement was disappointing across all measures.

Our study is though not without limitations. Not only have the samples of the trials conducted until now been small, but only 5/8 of the eligible trials could provide IPD. Including IPD from the three remaining trials^23–25^ is unlikely though to have changed our findings. They included only 72 (15.3%) of the 470 PWE randomised by the eight trials. The size of the treatment effect on depression in these trials also did not significantly differ from that in trials providing IPD.

The trials identified by our search used different outcome measures for distress. That they did this was not surprising.^48 49^ It needs to be acknowledged though that not all instruments are equal in their psychometric properties, including responsiveness to change. Moreover, there appears to be a lack of epilepsy-specific validity data for the context in which 3/9 outcome measures were used in by the trials^50 51^ (namely, the modified Beck Depression Inventory in the USA; Hopkins Symptom Checklist-20 in the USA and Beck  Depression Inventory in Germany). This possible lack of equivalence between the trials’ in their outcome measures should be considered when interpreting change seen between and across the trials in our review (though not within).

The trials in the review also reported limited follow-up data. We thus considered only the benefit of CBT apparent at the first post-treatment outcome assessment in the trial. Trials with longer term follow-up are therefore required to describe longer term utility. Such evaluations are important in view of evidence from the wider literature that ~30% of persons with depression relapse following CBT.^52–54^ In online supplementary table 4 we do present the proportion of PWE showing reliable improvement in the two trials,^11 27 28^ which included longer term follow-ups and for which IPD were received. The picture provided is mixed. On the primary depression outcome measure in Ciechanowski *et al*’s^27^ trial, the proportion reliably improving increased, but in Gandy *et al*’s trial it reduced.^11^ These findings should though be interpreted with caution as loss to follow-up was high and not always equal across treatment arms.

Finally, it may have been inappropriate to focus on the pooled RDs since the trials employed different modes of treatment delivery and had samples with different levels of pretreatment distress. Doing so may underestimate the ability of one of the treatments to elicit a reliable response, not least because participants with lower levels of depression have less scope to improve. This did not, however, appear to be the case. Ciechanowski *et al*’s^27^ trial, for example, had the most effective individual treatment with a RD of 0.26 and the pooled RD was only slightly lower at 0.20. We also presented separate pooled RDs for trials evaluating face-to-face individual CBT and for trials evaluating CBT delivered remotely and/or in groups. These indicated face-to-face individual CBT may lead to more improvement. The extent of change though continued to be modest, with only 29% of treated PWE reliably improving in such trials compared with controls. Moreover, the CI for the RD for this type of treatment overlapped with that of the pooled RD for the other types of CBT, raising the possibility that no difference exists between the approaches.

## Conclusions

Available CBT treatments have limited benefit for depressive symptoms in PWE. People receiving them *are* more likely to respond in the short term than if they receive usual care. However, *most* patients who receive the treatments do not show reliable improvement. The long-term effect of the treatments for depression remains to be determined, as does their benefit for anxiety. Overall, the results imply there is substantial scope for improvements in psychological treatments for distress in PWE. This may be found through exploring alternative psychotherapeutic approaches.
