# Supplementary material for: Cognitive-behavioural therapy does not meaningfully reduce depression in most people with epilepsy: a systematic review of clinically reliable improvement
Source: J Neurol Neurosurg Psychiatry. 2018 May 7;89(11):1129–37. doi: 10.1136/jnnp-2018-317997 (PMC6227812; doi:10.1136/jnnp-2018-317997)
Supplement: Supplementary file 1 [file jnnp-2018-317997supp001.pdf]

Supplementary files

Supplementary Table 1 Search terms

Medline, PsycINFO, PsycARTICLES, CINAHL plus, AHMED, clinicaltrials.gov, EThOS and SIGLE were searched from inception to March 2017 using Medical Subject Heading terms and keywords to identify suitable trials. Combinations of terms associated with psychological interventions, emotional distress, and epilepsy were used. The strategy below used for PsycINFO was adapted for each electronic database.

Order	Search term
1.	epilep*
2.	AND depress* OR anxi* OR mood OR 'quality of life'
3.	AND therap* OR interven* OR treat* OR rehab* OR psycho*OR cognitive*
4.	AND trial OR random*

To ensure a comprehensive search, reference lists were also hand-searched for additional studies.

Supplementary Table 2 Quality ratings for eligible trials providing individual patient data

Trial	PEDro Scale[13]											
	Criteria 1	*Criteria 2*	*Criteria 3*	*Criteria 4*	*Criteria 5*	*Criteria 6*	*Criteria 7*	*Criteria 8*	*Criteria 9*	*Criteria 10*	*Criteria 11*	**Total score**
	**Eligibility criteria specified**	**Subjects randomly allocated**	**Treatment allocation concealed**	**Groups similar at baseline**	**Blinding of subjects**	**Blinding of therapists**	**Blinding of assessors**	**Measures obtained from more than 85% of subjects initially allocated to groups**	**Subjects received treatment as allocated or data analysed by 'intention to treat'**	**Between-intervention group statistical comparisons reported**	**Point measures and measures of variability reported**	
**Thomspon et al.** 2010 [34]	Yes	Yes	Yes	Yes	No	No	No	No	No	Yes	Yes	5
**Ciechanowski et al.** 2010 [35]	Yes	Yes	Yes	Yes	No	No	Yes	No	Yes	Yes	Yes	7
**Schröeder et al.** 2014 [25]	Yes	Yes	Yes	Yes	No	No	Yes	No	Yes	Yes	Yes	7
**Gandy et al.** 2014 [24]	Yes	Yes	Yes	Yes	No	No	No	No	Yes	Yes	Yes	6
**Thompson et al.** 2015 [30]	Yes	Yes	Yes	Yes	No	No	No	No	Yes	Yes	Yes	6

Notes: Each item is scored as yes (1) or no (0). According to guidelines for this instrument, scores for items 2–11 are added to form an overall score: 9-10 indicates a methodologically "excellent" trial, 6-8 "good" quality, 4-5 "fair" and ≤3 "poor" quality.

Supplementary Table 3 Characteristics of eligible trials

**Thompson et al.**[26] (2010, U.S.A)	
Eligibility	*Inclusion criteria:* Aged ≥21 diagnosed with epilepsy for ≥1 year; mild-moderate depression (CES-D score 14-37).<br>*Exclusion criteria:* Suicidal ideation; cognitive impairment on screening test.
Treatment conditions	*Compared:* CBT+M to WLC. CBT+M: Group intervention comprising 8*60 min. weekly sessions delivered by phone or internet. Co-facilitated by lay person with epilepsy and research assistant. Supervised by clinical psychologist. *Receipt of intervention:* Mean CBT-M sessions completed by participants=6.5/8 (range 3-8).
Sample	*Recruitment:* 53 participants enrolled. 26 allocated to CBT+M, 27 to WLC. *Demographics:* Participants' mean age in CBT-M group 36.4 and 35 in WLC. 20 (77%) of CBT+M group and 23 (85%) of the WLC group were female. *Epilepsy:* 13 (50%) in CBT-M group and 19 (70.3) in WLC group had a seizure in prior 4 weeks. *Distress:* 10 (38.4%) in CBT-M and 9 (33.3%) in WLC had major depressive disorder. Mean CES-D score for CBT-M group was 25.7 and 27.33 for WL group.
Outcome assessment//s	*Timing:* ~8 weeks post-randomization. *Retention:* 19 (73.1%) CBT-M participants and 21 (77.7%) WLC participants completed assessments.
Outcome measure/s	*Primary:* Depression, mBDI[15]; *Secondary:* Depression, PHQ-9 [16]
**Ciechanowski et al.[27]** (2010, U.S.A).*	
Eligibility	*Inclusion criteria:* Persons aged ≥18, diagnosed with epilepsy according to ICD-9 and with clinically significant depression on PHQ-9 (>10) or dysthymia at interview. *Exclusion criteria:* Bipolar depression; receiving 'psychiatric treatment'; cognitive impairment on screening test.

Treatment conditions	*Compared:* CBT+M to TAU. CBT+M: Comprised 8*50 min. face-to-face sessions in homes delivered by social workers over 19-weeks. From week 19-52, monthly telephone monitoring calls attempted. Supervised by psychiatrist. *Receipt of intervention:* Mean CBT+M sessions completed 6.2/8; 29 (72.5%) CBT participants completed ≥6 sessions. *Note:* Supervising psychiatrist wrote to physician of CBT-M participants if no improvement shown. Psychiatric also wrote to physicians of TAU participants reporting depression and encouraging treatment.
Sample	*Recruitment:* 80 participants enrolled. 40 allocated to CBT+M, 40 to TAU. *Demographics:* Mean age of participants was 43.4 in CBT+M and 44 in the WLC. 19 (47.5%) of CBT+M participants and 23 (57.5%) of TAU participants were female. *Epilepsy:* 29 (72.5%) CBT+M participants and 30 (75.0) TAU participants had ≥1 seizure within prior 6 months. 19 (47.5%) CBT+M participants and 22 (55.0%) TAU participants had seizures involving a loss of consciousness (LOC). *Distress:* Mean baseline HSCL-20 score for CBT-M group was 2.1 and 1.9 for TAU group
Outcome assessment//s	*Timing:* 6, 12 and 18 months post randomisation. *Retention***:** Of CBT+M group, 32 (80%), 31 (77.5%) and 26 (65.0%) of participants completed respective assessments. Of the WLC participants, 33 (82.5%), 28 (70.0%) and 27 (67.5%) of participants completed them.
Outcome measure/s	*Primary:* Depression, HSCL-20 [17]

Schröder et al.[12] (2014, Germany)

Eligibility	*Inclusion criteria:* Persons aged ≥18 who self-reported an epilepsy diagnosis and experience of depression (no formal diagnosis required). *Exclusion:* Bipolar depression; suicidal ideation.
Treatment conditions	*Compared:* CBT+M+ACT to. WLC. CBT+M+ACT intervention was delivered to individual online. Comprised of 12 modules. Modules take 10–60 minutes and programme was accessible for 9 wks**.** *Receipt of intervention:* No information reported.

Sample	*Recruitment:* 78 participants enrolled, 38 allocated to CBT+M+ACT, 40 to WLC. *Demographics:* Participants mean age was 35.03 in the CBT+M+ACT group and 40.03 in the WLC group. 27 (71.1%) of CBT+M+ACT and 32 (80.0%) of WLC participants were female. *Epilepsy:* 26 (68.4%) of CBT group and 27 (67.5%) of the WLC group reported ≤1 seizure (type unspecified) a month. The remaining 12 (31.6%) CBT participants and 13 (32.5%) WLC participants experienced >1 seizure a month. *Distress:* On average, both groups had minimal–mild depressive symptoms (BDI ≤ 18) with an average BDI score of 22.2 in CBT+M+ACT group and 19.4 for WLC
Outcome assessment//s	*Timing:* ~9 weeks post randomisation. *Retention:* 25 (65.8%) of CBT+M+ACT group and 32 (80.0%) of WLC group completed assessments.
Outcome measure/s	*Primary:* Depression, BDI-I[18]

Gandy et al. [11] (2014, Australia)

Eligibility	*Inclusion criteria:* Persons aged 18-65 with neurologist confirmed epilepsy diagnosis according to ILAE [56] criteria.<br>*Exclusion criteria:* IQ score of <80; suicidal ideation.
Treatment conditions	*Compared:* CBT to WLC. CBT: Delivered face-to-face to individuals. Comprised of a 1-2-hr assessment followed by 8*60-minute weekly sessions delivered by psychology doctorate students within clinics, supervised by clinical psychologists. *Receipt of intervention:* 61.3% of participants completed all 9 CBT sessions.
Sample	*Recruitment:* 59 participants enrolled, 31 allocated to CBT, 28 to WLC. *Demographics:* Participants mean age 41 in CBT group and 38 in WLC group. 16 (50%) of CBT and 21 (76%) of WLC participants were female. *Epilepsy:* Mean years since diagnosis was 15 for CBT group and 12 for WLC. 14 (70%) CBT participants and 21 (79%) WLC participants had focal epilepsy. Remaining 6 (30%) CBT and 4 (16%) WLC participants had generalised epilepsy. 9 (45%) of the CBT participants and 13 (52%) of WLC participants were noted as having "refractory" epilepsy according to their neurologist (criteria used not given). *Distress:*

	Both CBT and WLC participants had a mean baseline NDDI-E score of 16. 17 (55%) of CBT group and 18 (64%) of WLC group had a score (≥15) indicating clinically significant depression.
Outcome assessment//s	*Timing:* ~9 and 12 weeks post randomisation. *Retention:* Of CBT participants, 20 (64.5%) and 19 (61.3%) completed assessments at respective time points. 25 (89.3%) and 23 (82.1%) WLC participants completed the assessments.
Outcome measure/s	*Primary:* Depression, NDDI-E [19]; *Secondary:*[†] Depression, HADS-D; Anxiety, HADS-A.[29]
**Thompson et al.[22]** (2015, U.S.A)	
Eligibility	*Inclusion criteria:* Persons aged ≥21 who had been diagnosed with epilepsy for ≥3 months and with mild-moderate depression (CES-D score 8-26). *Exclusion criteria:* Cognitive impairment on screening test.
Treatment conditions	*Compared:* CBT+M to WLC. CBT+M: Group intervention comprising 8*60-minute weekly sessions delivered by phone or internet by a lay person with epilepsy and a graduate student, supervised by clinical psychologist. *Receipt of intervention:* Mean CBT-M sessions completed 6.6/8. *Note:* WLC participants 'contacted' weekly by study facilitators to control for interaction with project staff.
Sample	*Recruitment:* 128 participants enrolled, 64 allocated to CBT, 64 to WLC. *Demographics:* Baseline demographics reported for groups combined and after exclusion of 10 randomised participants identified as ineligible. Mean age was 41.2 and 77 (65.3%) were female. *Epilepsy:* No further details reported. *Distress:* Mean baseline mBDI score for CBT group was 20.3 and 20.2 for WLC group.
Outcome assessment//s	*Timing:* ~10.5 weeks post randomisation. *Retention:* 52 (81.3%) of CBT+M group and 56 (87.5%) of the WLC participants completed assessment.
Outcome measure/s	*Primary:* Depression, mBDI [15]; *Secondary:* Depression, PHQ-9[16]; NDDI-E.[19]
**Davis et al.[23]** (1984, U.S.A)	

Eligibility	*Inclusion criteria:* Stated participants were ‘depressed’ individuals with diagnosed epilepsy prescribed AEDs, who responded to trial adverts.<br>*Exclusion criteria:* IQ<70; “behaviour problems that precluded participation”.
Treatment conditions	*Compared:* CBT to WLC. CBT: Group intervention comprising 6*120 minute weekly face-to-face sessions facilitated by 2 social workers. Six weeks after final session, review session occurred. *Receipt of intervention:* No information reported.
Sample	*Recruitmen***t:** 15 participants enrolled, 8 allocated to CBT, 7 to WLC. *Demographics:* Mean age for CBT group was 33.5; and 7 (87.5%) of its participants were female. For WLC group, only the characteristics of 5 of completed outcome assessment were reported. Their mean age was 32.4; 3 (60.0%) were female. *Epilepsy:* For entire sample the mean years since diagnosis was 13.69. “Most” participants said to have ≥1 seizure p/week. Distribution of seizure types, time since diagnosis, and seizure frequency reported as comparable between groups. *Distress:* Mean BDI-I score was the same for CBT and WLC groups at 20.75; 6 (75%) of the CBT group and 5 (100%) of the WLC group had a score (≥8) indicating clinically significant depression
Outcome assessment//s	*Timing:* 6 weeks post-randomisation. *Retention:* 8 (100%) of CBT group and 5 (71.4%) of WLC participants completed assessment.
Outcome measure/s	*Primary:* Depression, BDI-I.[18]; *Secondary:* Depression, DACL; Hudson GCS.
**Tan & Bruni** [24] (1986, U.S.A) ††	
Eligibility	*Inclusion criteria:* Stated ‘adults’ with neurologist confirmed epilepsy diagnosis referred to study with “significant psychosocial problems and inadequate seizure control.” *Exclusion criteria:* “Mental retardation”; psychosis.
Treatment conditions	*Compared:* CBT to WLC. CBT: Group intervention comprising of 8*120 minute weekly face-to-face sessions, delivered by a clinical psychologist. *Receipt of intervention:* CBT participants attended an average of 7.4/8 session.

Sample	*Recruitment:* 20 participants enrolled, 10 allocated to CBT, 10 to WLC. *Demographics:* 6 (62.5%) CBT participants and 7 (67%) WLC participants were female. Baseline mean age provided only for sample completing outcome assessment. It was 33.4. *Epilepsy:* Mean years since diagnosis was reported for total sample as 15.5 and said to be comparable between groups. *Distress:* Mean BDI score for CBT group 11.13, for the WLC group it was 12.00.
Outcome assessment//s	*Timing:* 8 and 16 weeks post-randomisation. *Retention:* 8 (80%) CBT participants and 9 (90%) WLC participants completed 8-week assessment. 8 (80%) CBT participants and 9 (90%) of WLC participants completed 16-week assessment.
Outcome measure/s	*Primary:* Depression, BDI-I.
**McLaughlin & McFarland [25]** (2011, Australia)	
**Eligibility**	*Inclusion criteria:* People aged ≥60; with a doctor confirmed epilepsy diagnosis. *Exclusion criteria:* Cognitive impairment on screening test.
**Treatment conditions**	*Compared*: CBT to RT. CBT: Group intervention comprising of 6*120 minute weekly face-to-face sessions delivered by a clinical psychologist. RT: Comprised of group relaxation training, 6*60 minute weekly face-to-face. Training of facilitator not specified. *Receipt of intervention:* No information reported
**Sample**	*Recruitment:* 37 participants enrolled, 18 allocated to CBT, 19 to RT. *Demographics:* Participants mean age was 67.5 in CBT group and 67.3 in RT group. 10 (56%) of CBT and 9 (47%) of RT participants were female. *Epilepsy:* Mean years since diagnosis for CBT group was 28.56 compared to 25.89 in RT group. In CBT group, 8 (44.4%) participants had generalized seizures, and 10 (52.6%) partial. In the RT group, 9 (47.3%) had generalized seizures, and 10 (52.6%) partial. *Distress:* Mean GDS score for CBT group 12.50, compared to 11.37 for RT group
**Outcome assessment//s**	*Timing:* 8 and ~18 weeks post-randomisation. *Retention:* 100% of CBT group and 100% of RT group completed both assessments.
**Outcome measure/s**	*Primary:* Depression, GDS.

Notes: ACT, Acceptance and Commitment Therapy; BDI, Beck Depression Inventory; CBT, cognitive behavioural therapy, CES-D, Center for Epidemiological Study of Depression measure; DACL, Depression Adjective Checklist form E; FU, follow-up; GCS, Hudson Generalized Contentment Scale; GDS, Geriatric Depression Scale; HADS-A, Hospital Anxiety and Depression Scale –Anxiety subscale; HADS-D, Hospital Anxiety and Depression Scale – Depression subscale; HSCL-20, Hopkins Symptom Checklist-

20; ICD-9, International Classification of Diseases Ninth Revision; ILAE, International League Against Epilepsy; IPD, individual patient data; M, mindfulness; mBDI, Modified Beck Depression Inventory; mo., month; NDDI-E, Neurological Disorders Depression Inventory for Epilepsy; PHQ-9, Patient Health Questionnaire-9; RT, relaxation training; SD, standard deviation; TAU, treatment as usual; wk., week; WLC ,waitlist control.
* 18 month follow-up reported by Chaytor et al. (2011)
† The designation of outcome measures as being the primary or secondary outcome measure was taken directly from trial reports, except for Gandy et al. (2014) (see section 3.1.4)
†† Tan and Bruni study also included an active control condition, namely Supportive Counselling. The effect of the CBT against this condition is not reported here.

Supplementary Table 4 Classification of change in participants' psychological distress between baseline and additional follow-up assessments in Ciechanowski et al. [35] and Gandy et al.'s [24] trials according to Jacobson's Reliable Change Index

Trial	Follow-up assessment 2**				Follow-up assessment 3***			
	Reliable Change category (%)				Reliable Change category (%)			
	n	*Unchanged*	*Deteriorated*	*Improved*	*n*	*Unchanged*	*Deteriorated*	*Improved*
**Ciechanowski et al.** 2010 [27]								
Primary HSCL-20								
CBT	31	45.2	3.2	51.6	26	26.9	7.7	65.4
TAU	28	85.7	3.6	10.7	27	74.1	11.1	14.8
		Difference % improved relative to control= 40.9				Difference % improved relative to control= 50.6		
**Gandy et al.** 2014 [11 †]								
Primary: NDDI-E								
CBT	19	73.7	0	26.3				
WLC	23	78.3	8.7	13.0				
		Difference % improved relative to control= 13.3						
Secondary: HADS-D								
CBT	19	73.7	10.5	15.8				
WLC	23	82.6	13.0	4.3				
		Difference % improved relative to control= 11.5						
Secondary: HADS-A								
CBT	19	63.2	5.3	31.6				
WLC	23	87.0	8.7	4.3				
		Difference % improved relative to control= 27.3						

Notes: Timing of assessments were: Ciechanowski et al. (2010) follow-up assessment 1 ("post-treatment assessment")= 6 months, follow-up assessment 2= 12 months; follow-up assessment 3= 18 months (reported within Chaytor et al., 2011) ; Gandy et al. (2014) follow-up assessment 1 ("post-treatment assessment")= 9 weeks, follow-up assessment 2= CBT, cognitive behavioural therapy; HADS-A, Hospital Anxiety and Depression Scale –Anxiety subscale; HADS-D, Hospital Anxiety and Depression Scale – Depression subscale; HSCL-20, Hopkins Symptom Checklist-20; NDDI-E, Neurological Disorders Depression Inventory for Epilepsy; WLC ,waitlist control. † The designation of outcome measures as being the primary or secondary outcome measure was taken directly from trial reports, except for Gandy et al.(2014) (see section 3.1.4).

Supplementary Figure 1

Difference in proportion of participants showing "reliable improvement" within the eligible trials on primary outcomes of depression post-treatment according to whether treatment was individual face-to-face CBT or of another form

Group by Face-to-face, individual CBT?	Study name	Risk difference	Lower limit	Upper limit	Tx	Control	p-Value	Relative weight
No	Thompson et al. 2010	0.08	-0.20	0.35	6 / 19	5 / 21	0.58	11.23
No	Schroder et al. 2014	0.25	0.06	0.43	7 / 25	1 / 32	0.01	24.94
No	Thompson et al. 2015	0.19	0.08	0.31	11 / 52	1 / 56	0.00	63.83
No		0.19	0.10	0.29			0.00	
Yes	Ciechanowski et al. 2010	0.26	0.03	0.48	16 / 32	8 / 33	0.03	47.94
Yes	Gandy et al. 2014	0.17	-0.05	0.39	5 / 20	2 / 25	0.13	52.06
Yes		0.21	0.06	0.37			0.01	
Overall		0.20	0.12	0.28			0.00	

Statistics for each study

Risk difference and 95% CI

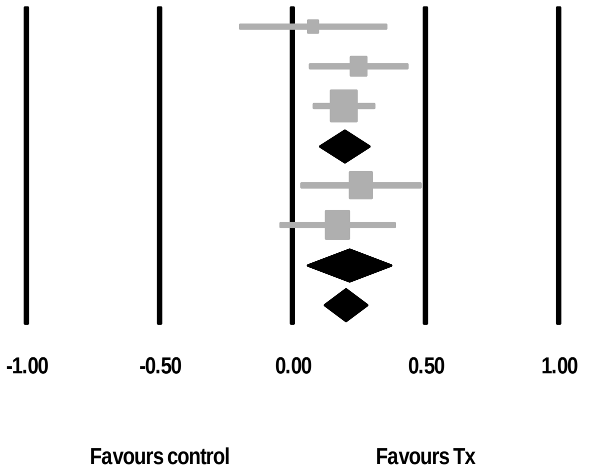

NOTES: Duration of intervention, timing of post-treatment assessment, and primary outcome measure used were: Thompson et al. (2010) intervention duration= 8 weeks, timing of post-treatment assessment= ~8 weeks, measure= Modified Beck Depression Inventory (mBDI-I); Ciechanowski et al.(2010) intervention duration 19 weeks, timing of post-treatment assessment=24 weeks, measure= Hopkins Symptom Checklist-20 (HSCL-20); Schröder et al. (2014) intervention duration= 9 weeks, timing of post-treatment assessment= 9 weeks, measure=Beck Depression Inventory (BDI-I); Gandy et al. (2014) intervention duration= 8 weeks, timing of post-treatment assessment= 9 weeks, measure=Neurological Disorders Depression Inventory for Epilepsy (NDDI-E); Thompson et al.(2015) intervention duration= 8 weeks, timing of post-treatment assessment= 10.5 weeks, measure= mBDI,; † The designation of outcome measures as being the primary or secondary outcome measure was taken directly from trial reports, except for Gandy et al. (2014) (see section 3.1.4)

Supplementary Figure 2

When participants without clinical distress at baseline are excluded, difference in proportion of participants showing "reliable improvement" within the eligible trials on primary outcomes of depression post-treatment according to whether treatment was individual face-to-face CBT or of another form

Group by Face-to-face, individual CBT?	Study name	Risk difference	Lower limit	Upper limit	Tx	Control	p-Value	Relative weight
No	Thompson et al. 2010	0.08	-0.20	0.35	6 / 19	5 / 21	0.58	12.41
No	Schroder et al. 2014	0.17	-0.07	0.41	7 / 22	4 / 27	0.16	17.08
No	Thompson et al. 2015	0.19	0.08	0.31	11 / 52	1 / 56	0.00	70.51
No		0.18	0.08	0.27			0.00	
Yes	Ciechanowski et al. 2010	0.26	0.03	0.48	16 / 32	8 / 33	0.03	70.56
Yes	Gandy et al. 2014	0.36	0.01	0.71	6 / 11	3 / 16	0.05	29.44
Yes		0.29	0.10	0.48			0.00	
Overall		0.20	0.11	0.29			0.00	

Statistics for each study; Risk difference and 95% CI

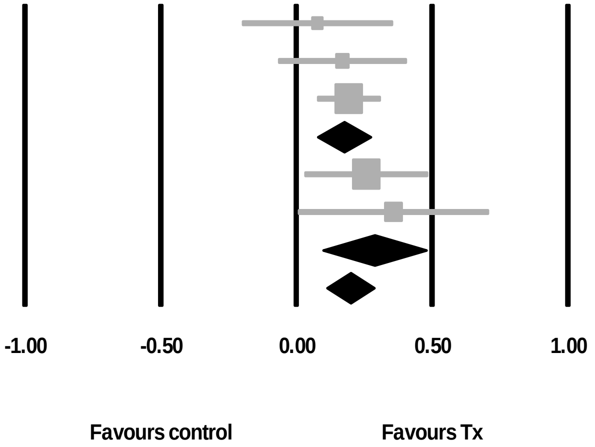

NOTES: There were some participants without clinically significant distress at baseline in Schröder et al.'s (2014) (n=3 intervention and n=5 control participants) and Gandy et al.'s (2014) (n=9 intervention and n=9 control participants) trials. The exclusion of these participants meant the individual RCIs for these two trials needed to be recalculated as the SD of the trial samples on the primary outcome measure of interest for the trial changed. The recalculated RCI was 9.23 for Schröder et al. (2014), and 2.41 for Gandy et al. (2014).

Duration of intervention, timing of post-treatment assessment, and primary outcome measure used were: Thompson et al. (2010) intervention duration= 8 weeks, timing of post-treatment assessment= ~8 weeks, measure= Modified Beck Depression Inventory (mBDI-I); Ciechanowski et al.(2010) intervention duration 19 weeks, timing of post-treatment assessment=24 weeks, measure= Hopkins Symptom Checklist-20 (HSCL-20); Schröder et al. (2014) intervention duration= 9 weeks, timing of post-treatment assessment= 9 weeks, measure=Beck Depression Inventory (BDI-I); Gandy et al. (2014) intervention duration= 8 weeks, timing of post-treatment assessment= 9 weeks, measure=Neurological Disorders Depression Inventory for Epilepsy (NDDI-E); Thompson et al.(2015) intervention duration= 8 weeks, timing of post-treatment assessment= 10.5 weeks, measure= mBDI,; † The designation of outcome measures as being the primary or secondary outcome measure was taken directly from trial reports, except for Gandy et al. (2014) (see section 3.1.4)
